# Health related quality of life (HRQOL) of patients with End Stage Kidney Disease (ESKD) on hemodialysis in Addis Ababa, Ethiopia: a cross-sectional study

**DOI:** 10.1186/s12882-021-02494-9

**Published:** 2021-08-16

**Authors:** Sujin Kim, Yemisrach Nigatu, Tekebash Araya, Zewdu Assefa, Nebiyu Dereje

**Affiliations:** 1 Department of Medicine, Myungsung Medical College, Myunsung Christian Medical Center, Addis Ababa, Ethiopia; 2 Department of Public Health, Myungsung Medical College, Myunsung Christian Medical Center, P.O Box 15478, Addis Ababa, Ethiopia; 3 Department of Nephrology, Myungsung Medical College, Myunsung Christian Medical Center, Addis Ababa, Ethiopia

**Keywords:** ESKD, HRQOL, Hemodialysis, CKD, Ethiopia

## Abstract

**Background:**

End-Stage Kidney Disease, the most severe form of chronic kidney disease, is fatal if not treated by renal replacement therapy. Thus, patients with End-Stage Kidney Disease depend on hemodialysis as a lifesaving treatment for the remainder of their lives. However, the health-related quality of life (HRQOL) of patients on hemodialysis is much more underappreciated in resource limited countries.

**Methods:**

A hospital based cross-sectional study was conducted in Addis Ababa, Ethiopia, from August 01 to October 31, 2019. All patients who were on hemodialysis in five randomly selected public and private hospitals (n = 125) were included in the study. Data were administered by trained nurses by using a standardized Kidney Disease Quality of Life questionnaire. Clinical data were extracted from the patients’ medical charts. HRQOL was categorized as low, if the overall mean score was ≤ 50, or as high, if the overall mean score was > 50. Factors associated with lower HRQOL were identified by multi-variable binary logistic regression analysis and expressed by adjusted odds ratio (aOR) and its respective 95 % confidence interval.

**Results:**

The mean age of the study participants (n = 125) was 50.33 years (± 15.07) and more than two-thirds (68.8 %) of the participants were males. The mean score of HRQOL was 49.08 ± 11.09, with 48.0 % (95 % CI: 42.2 − 54.5 %) of them had lower HRQOL. Unemployed patients (aOR = 2.40, 95 % CI: 1.10–5.90) and patients who had hemodialysis 2 times per week (aOR = 1.71, 95 % CI: 1.07–3.83) had lower HRQOL. Elderly patients had higher odds of having lower mean score on the burden of kidney disease (aOR = 2.07; 95 % CI 1.18–4.13) as compared to the younger patients.

**Conclusions:**

Nearly half of the patients with ESKD on hemodialysis had lower overall HRQOL which is associated with their unemployment status and frequency of hemodialysis per week. Elderly patients had lower mean score of burden of kidney disease. Therefore, quality of life of patients with chronic dialysis should be given special attention during the patients’ care. Measures should be taken by the government to ensure accessibility and affordability of the hemodialysis services in the country.

## Background

Chronic kidney disease (CKD) includes 5 stages which are categorized based on glomerular filtration rate (GFR) and albuminuria. End Stage Kidney Disease (ESKD) is a stage 5 of CKD (GFR < 15 ml/min/1.73 m^2^), characterized by accumulation of toxins, electrolytes, and fluid, resulting in uremia [1]. These patients depend on dialysis as a lifesaving treatment for the remainder of their lives unless they receive kidney replacement therapy [2].

Globally, there were 697.5 million cases of CKD (the prevalence of 9.1 %) and 1.2 million people died from CKD in 2017. In Ethiopia, the prevalence of CKD was 4.6 % and 9083 patients died due to CKD in 2017 [3].

Patients on hemodialysis (HD) has double burden from CKD and HD. CKD itself has negative effects on HRQOL, and the quality of life progressively decreases with the advanced disease [4]. Moreover, HRQOL has been shown to be associated with increased mortality in patients with ESKD [5, 6]. Furthermore, since HD is time-intensive, expensive and requires fluid and dietary restrictions; the physical, psychological, socioeconomic, and environmental aspects of life are negatively affected—leading to compromised quality of life for the patients on HD [7, 8].

It is estimated that only 4000 patients with ESKD are on HD in Sub-Saharan Africa (SSA), which is less than 1 % of the global cases on HD [9]. However, HD for ESKD do not only prolong life but also sustain quality of life [10, 11]. Since HRQOL is related with the morbidity and mortality among HD patients, it is suggested that HRQOL should be considered in the regular monitoring of HD patients [12]. In Ethiopia, there is only one study which was conducted among CKD patients, showing quality of life was decreased in all stages of CKD [8]; however, to our knowledge, there is no study done on patients with ESKD on hemodialysis. The extent of HRQOL among patients with ESKD on hemodialysis in Ethiopia is not known. Therefore, this study is aimed to assess the magnitude and associated factors of Health-Related Quality of Life (HRQOL) among ESKD patients on hemodialysis in Addis Ababa, Ethiopia.

## Methods

 All methods were carried out in accordance with relevant guidelines and regulations.

### Study setting, design, and population

A hospital based cross-sectional study was conducted from August 01 to October 31, 2019, among adult patients on hemodialysis in selected dialysis units in Addis Ababa, Ethiopia. Addis Ababa is a capital city of Ethiopia, with an estimated population of 4.5 million (according to the projections based on 2007 census). There were 5 public and 25 private hospitals in Addis Ababa during the study period. Out of which, ten of them were providing hemodialysis service. The dialysis units (hospitals) included in this study were three private hospitals (Myungsung General Hospital, Bethzatha Hospital and Addis-Hiwot hospital) and two public hospitals (Zewditu Memorial Hospital and Menelik II Hospital). All the patients with ESKD (n = 125) who were on hemodialysis in the selected hospitals during the study period were included in the study.

### Data collection tools and procedures

A structured standardized Kidney Disease Quality of Life (KDQOL™-36) questionnaire was used. The KDQOL™-36 questionnaire contains 5 subscales: the first 2 subscales (Physical Component Summary (PCS) and Mental Component Summary (MCS)) are in the Items 1–12, Burden of Kidney Disease (BKD) for items 13–16, Symptoms and Problems of Kidney Disease (SPKD) for items 17–28, and Effects of Kidney Disease (EKD) for items 29–36. The first two subscales are a generic measure of HRQOL whereas the last three assess issues specific to patients with ESKD or earlier stages of chronic kidney disease [6–8].

Even though KDQOL™-36 is designed for self – administered questionnaire, as some of the patients in the research setting may not be able to read and write, we used KDQOL™-36 as an interview-based questionnaire for the better quality of data. The Amharic translated version of the questionnaire (KDQOL™-36) was used by data-collectors who can speak Amharic. The questionnaire was crosschecked by experts in the field to check whether the questionnaire was matched to the context. Biochemical profile such as level of hemoglobin, blood urea nitrogen and creatinine and clinical profile such as the number and types of comorbidities, duration of dialysis, and frequency of dialysis were extracted from the patients’ medical charts.

### Data management and analysis

Data entry and statistical analysis were performed using SPSS 25th version. Data were summarized as mean and standard deviation (SD) for the continuous variables, and frequency and percentage for categorical variables.

The HRQOL was determined using the KDQOL-36™scoring program (V2.0), which is provided as a program in an Excel spreadsheet used to create scores and produce descriptive statistics for the KDQOL-36™ measures [11]. The scores were constructed using the Likert method of summated ratings. Answers to each question were scored and then summarized to produce raw scale scores for each health concept, and thereafter transformed to a 0-100 scale with higher scores indicating better quality of life [12].

The main outcome variable of the study was the overall HRQOL and five subscales of the KDQOL-36™ (PCS, MCS, BKD, SPKD and EKD) were presented as the mean score and standard deviation. The overall mean score ≤ 50 and > 50 was considered as lower and higher HRQOL, respectively [7, 8]. The exposure variables of the study include age, sex, marital status, educational status, occupation, monthly income, duration, and frequency of hemodialysis. Age was dichotomized as below or above the mean age. Educational status was dichotomized as illiterate (unable to read and write) or literate (able to read and write). Occupation was categorized as employed (currently working) and unemployed (currently not working). Comorbidity was dichotomized as yes or no comorbidity. Bivariate and multivariable binary logistic regressions were computed to identify associating factors with the HRQOL and expressed by adjusted odds ratio (aOR) with 95 % confidence interval (CI). Variables with a *P*-value < 0. 25 in bivariate analysis and those variables with clinical relevance were entered into multivariable binary logistic regression. The level of significant was set at 5 % and *P*-value < 0.05 was considered statistically significant. The goodness of model was assessed by the Hosmer Lemeshow test, and it was not significant (*P*-value = 0.83) and there was no multicollinearity (Variance inflation factor = 2.5 and the tolerance test = 0.4).

## Results

### Socio-demographic characteristics

All the 125 patients’ data were analyzed, with 100 % response rate. The mean age of the patients was 50.33 years (± 15.07), with about half of them (48.0 %) were older than 50 years. More than two-thirds of the patients (69.4 %) were males (Table 1).

**Table. 1:** Sociodemographic and baseline clinical characteristics of patients with ESKD on hemodialysis in Addis Ababa, 2019 (*n* = 125)

**Variables**	**Frequency**	**Percentage**
**Age in years**		
≤50	65	52.0
>50	60	48.0
**Sex**		
Male	86	68.8
Female	39	31.2
**Marital status**		
Single	29	23.3
Married	81	64.8
Divorced	11	8.8
Widowed	4	3.2
**Educational status**		
Illiterate	9	7.2
Literate	116	92.8
**Occupation**		
Employed	30	24.0
Unemployed	95	76.0
**Monthly income (Ethiopian Birr)**		
≤5000 (≤$150)	105	84.0
>5000 (>$150)	20	16.0
**Comorbidity**		
No	33	26.4
Yes	92	73.6
**Duration of hemodialysis (years)**		
≤3	99	79.2
>3	26	20.8
**Frequency of hemodialysis per week**		
2 times	39	31.2
3 times	86	68.8

### Health-Related Quality of Life – KDQOL-36™

The overall mean HRQOL was 49. 1 (± 11.1), with nearly half of the patients (48.0 %, 95 % CI: 42.2 − 54.5 %) had low HRQOL score. From the sub-scales, the lowest mean score (24.3 ± 22.7) was on the burden of kidney disease (BKD). A considerable proportion of the patients scoring below the reference value of 50 points (scale from 1 to 100 points) was found in three of the five KDQOL-36™ subscales (Table 2).

**Table. 2:** Health-related quality of life scores in subscales in Addis Ababa, 2019

Sub-scales	Mean ± SD	Minimum	Maximum	Score	Number	Percentage
**Symptom/problem list**	79.7 ± 14.3	31.2	100	< 50	4	3.2
				> 50	121	96.8
**Effects of kidney disease (EKD)**	58.5 ± 20.0	9.4	96.8	< 50	40	32.0
				> 50	85	68.0
**Burden of kidney disease (BKD)**	24.3 ± 22.7	0	81.2	< 50	101	80.8
				> 50	24	19.2
**SF-12 PCS**	36.6 ± 10.0	18.6	57.8	< 50	109	87.2
				> 50	16	12.8
**SF-12 MCS**	46.4 ± 11.2	57.8	66.7	< 50	72	57.6
				> 50	53	42.4

### Factors associated with lower HRQOL score

Those variables with *p* - value < 0.25 in the bivariate analysis and variables with clinical relevance were included in the multivariable analysis. These variables include age, sex, marital status, occupation, monthly income, comorbidity, duration of the hemodialysis and frequency of the hemodialysis per week. However, in the multivariable model (Table 3), only occupation and comorbidity were found to be significantly associated with lower HRQOL.

**Table 3:** Multivariable analysis showing factors associated with HRQOL among patients with ESKD in Addis Ababa, Ethiopia, 2019

**Variables**	**HRQOL score**		**cOR(95% CI)**	**aOR(95% CI)**	***P*-value**
	**>50**	**≤50**			
**Age in years**					
≤50	32	33	1.26(0.62-2.54)	0.88(0.39-1.99)	0.76
>50	33	27	1.00	1.00	
**Sex**					
Male	48	38	1.00	1.00	0.19
Female	17	22	1.63(0.76-3.51)	1.75(0.75-4.06)	
**Marital status**					
Living with spouse	45	36	1.00	1.00	0.43
Not living with spouse	20	24	1.50(0.72-3.14)	1.40(0.61-3.19)	
**Occupation**					
Employed	12	18	1.89(0.82-4.36)	2.40(1.10-5.90)	0.03
Not employed	53	42	1.00	1.00	
**Monthly income**					
≤3200 ETB (≤US$100)	56	49	1.40(0.53-3.65)	1.36(0.51-3.77)	0.63
>3200 ETB (>US$100)	9	11	1.00	1.00	
**Comorbidity**					
No	14	19	1.69(0.78-3.77)	1.72(0.69-4.26)	0.24
Yes	51	41	1.00	1.00	
**Duration of hemodialysis**					
≤3 years	51	48	1.10(0.46-2.61)	0.97(0.38-2.46)	0.95
>3 years	14	12	1.00	1.00	
**Frequency of hemodialysis/week**					
2 times	17	22	1.64(0.76-3.51)	1.71(1.07-3.83)	0.04
3 times	48	38	1.00	1.00	

*ETB* Ethiopian Birr; *cOR* Crude Odds Ratio; *aOR* Adjusted Odds Ratio

The odds of lower HRQOL was two times (AOR = 2.40, 95 % CI: 1.10–5.90) more likely among unemployed patients than those patients who were employed and two times (AOR = 1.71, 95 % CI: 1.07–3.83) higher among those patients who had hemodialysis 2 times per week as compared to those who had 3 times per week.

In the further sub-group analysis, by including each sub-scale as a dependent variable in the multivariable analysis, the odds of having lower BKD score among elderly patients (> 50 years) was two times higher (AOR = 2.07; 95 % CI 1.18–4.13) as compared to the younger patients (≤ 50 years) on hemodialysis. Moreover, the odds of having lower BKD score among those who had hemodialysis 2 times/week was two times higher (AOR = 2.13; 95 % CI 1.21–5.04) as compared to those patients who had hemodialysis 3 times/week. Other variables were not significantly associated with the subscales of the HRQOL.

## Discussion

Although there have been several studies about quality of life in patients on hemodialysis globally, only few studies have been conducted in the Sub-Saharan Africa (SSA). It has been suggested that nephrologists must look not only at the biological outcomes but also at the patient’s perceptions of their quality of life to accurately assess patient status [13]. Thus, the use of measuring HRQOL as a primary outcome of various interventions in ESKD treatment regimens is increasingly being accepted [14].

The principal findings of this study were that a substantial proportion of the patients with ESKD on hemodialysis in Addis Ababa, Ethiopia had lower HRQOL. From the subscales, consistent with the study findings from Chile [15], the patients had lower mean scores in the PCS (37.6), SF-12 (31.9), and MCS (43.5). The highest mean scores were observed on the BKD (56.9) and Symptom/Problem list (74.6) subscales which are consistent with previous studies conducted in Kenya, India, Chile, and Malaysia [13, 15–18]. The lower HRQoL score among the patients has several clinical implications. As HRQoL is one of the indicators of clinical outcome of the patients, the lower scores may indicate poor prognosis of the patients and compromised quality of clinical care. The findings of this study underscore that there is a need to assess the patients’ quality of life during the follow up and appropriate measures have to be taken to reassure the patients.

Consistent with previous studies conducted in Chile, US, Mangalore, Malaysia, and Korea [15–19], patients on hemodialysis in this study showed higher scores in MCS than in PCS. This means, despite the worsening of the physical health status, the mental health of dialysis patients is relatively preserved. This may reflect the ability of ESKD patients to adapt psychologically to their situation over time. Moreover, consistent to the study conducted in Netherlands [20], it was found that in patients who stayed on their initial dialysis modality, the physical QOL decreased over time, whereas the mental QOL tended to remain stable.

HRQOL was found to be associated with unemployment status of the patients and frequency of the hemodialysis. Similar findings have been also reported by previously conducted studies in Chile, US, and Mangalore [15–17]. The findings of this association might imply that financial hardships to cover the expenses of hemodialysis could have attributed to the lower HRQOL. In the further subgroup analysis, older age (> 50 years) patients had higher BKD compared to the younger patients. The subscale of BKD indicates that how much kidney disease interfere with daily life about time taking, frustration and making the respondent feel like a burden [11]. Thus, this finding showed that the kidney disease is more burdensome for the elderly patients on maintenance dialysis, compared to younger patients. This finding is consistent with many previous studies done in UAE, Chile, Romania, Korea, and India [15, 17, 19–21]. This finding underscores that there is need for special attentions for the care and reassurance of the elderly patients Moreover, the present study showed that patients with 2 times of hemodialysis per week were more likely to have lower HRQOL in Burden of Kidney disease. It is in the same line that 3 times weekly hemodialysis has been standard way of treatment to control uremia [22–26]. Due to the lack of access and affordability of hemodialysis in Ethiopia, many of the patients with ESKD may not get the service. As the result, patients take sub-optimal frequency of HD and suffer of the burden of the disease. For the better outcomes of the patients with ESKD, ensuring the access and affordability of the HD services in the city and the country is paramount critical.

To our knowledge, while there are few studies done for all stages of CKD patients, this study specifically targeted only for ESKD patients on maintenance dialysis as a first time in Ethiopia. Besides, this study was done in both private and governmental (public) hospitals where the payment of dialysis has huge difference. By covering both types of hospitals, it can show relatively generalized information possibly from the people who can afford hemodialysis payment without difficulty for the private hospital to the people who has had difficulty to pay for the governmental hospital. We have used a standard tool (KDQOL-36™ ) to assess the HRQOL in our study, which is useful to make international comparisons of our findings. However, this study could be limited in several regards. The sample size included in the study was minimal and might be underpowered to detect associations. Furthermore, there are some missing variables which has been known as important factors for dialysis patients such as Calcium, Phosphorus level and dialysate flow rate on Kt/V, which might give us additional information for the chronic dialysis patients. Due to the nature of interview-based data collecting, there might be reporting bias by data collectors or social desirability bias and recall bias by the patients.

In conclusion, this study showed that a substantial proportion of the patients with ESKD on hemodialysis in Addis Ababa had lower overall HRQOL which associated with their unemployment status and frequency of hemodialysis per week. Elderly patients had lower mean score of burden of kidney disease. Therefore, not only clinical treatment but also quality of life of patients with chronic dialysis should be given special attention during the hemodialysis patients’ care. Measures should be taken by the government to ensure accessibility and affordability of the hemodialysis services in the country.

## Data Availability

Data are available upon reasonable request from the corresponding author.

## Abbreviations

aOR: Adjusted Odds Ratio
BKD: Burden of Kidney Disease
CI: Confidence Interval
CKD: Chronic Kidney Disease
cOR: Crude Odds Ratio
EKD: Effects of Kidney Disease
ESKD: End Stage Kidney Disease
ETB: Ethiopian Birr
HRQOL: Health Related Quality of Life
KDQOL-36™: Kidney Disease Quality of Life
MCS: Mental Component Summary
OR: Odds Ratio
PCS: Physical Component Summary
QOL: Quality of Life
SD: Standard Deviation
SPKD: Symptoms and Problems of Kidney Disease
SPSS: Statistical Packages for Social sciences
